# The Rights of Babes to Be Well Born

**Published:** 1913-02

**Authors:** 


### The Rights of Babes to be Well Born.

We know that through all historical periods wrongs have been endured, but in the end have been resisted by the wronged, and have been righted, or are being righted. The Pilgrim Fathers faced the dangers of a savage foe and the hardships of the wilderness that they might exercise their right to worship God according to the dictates of their conscience. Our forefathers of the American Revolution took up arms in defense of their rights of citizenship. The French Revolution was a demand for the Rights of Man, written in blood.

We hear much of "Women's Rights," of the "Rights of Labor," and will hear more, for these are able to speak for themselves, and are speaking in voices that can and will be heard.

But there is a class that through the ages have suffered cruel wrongs,—who cannot, with audible voice, speak for themselves. This class is the *unborn,* whose wrongs in some respects have not been ameliorated, but in many things have been aggravated by civilization. They mutely cry to us, and stretch unseen hands for aid, and the motherhood of America will respond; yea, is responding to their appeal.

Every normal woman longs for motherhood; for the little one that sucks its life from her breast; for the little arms that twine about her neck; for the loving eyes that look confidingly into hers, and for the prattle of infant lips, sweeter to a mother's ear than the songs of the angel Israfel, whose heartstrings are a lute.

But, oh! the sorrow of the mother who sees her little one a physical wreck, a mental imbecile, or, learns later, that it is a moral pervert. If such a child could realize the miserable inheritance to which it is born, it might well exclaim with Job: "Let the day perish wherein I was born."

To the extent that such births are preventable, children have the right to be well born.

By being well born is not meant being born into wealth and culture. We cannot know whether such things will prove a blessing or a curse to a child. Wealth may have been obtained by greed that has cankered the soul. Culture may be of the head only; while the heart is ignorant of the things that make life blessed.

> "The guinea's but the stamp of rank;—
> A man's a man for a' that."

But a child has the right to have for a father a man that is a man, and for a mother a woman who is truly a woman. Jefferson wrote in the Declaration of Independence that "all men are created equal," by which he meant that the natural birthright of every man was political freedom and equality before the law.

Mothers of America, let us adopt a new Declaration of Independence, and declare that the natural birthright of every child born into the world is freedom from inheritable diseases—physical, mental or moral.

To what extent are physical ailments, mental weakness and moral degeneracy attributable to inheritance? Ask your family physician why your darling is blind. Ask the superintendents of insane asylums why the hopeless melancholy, the jibbering idiocy, the wild delusions, or the homicidal mania of many of those entrusted to their care. And when they have told you the awful truth you will find that it is but a paraphrase of what long ago was written in the Scriptures: "The fathers have eaten sour grapes and the children's teeth are on edge." Science confirms the statement that "the sins of the fathers (and of the mothers) are visited on the children to the third and fourth generation." Both Darwin and Morel say that the families of drunkards do not descend below the fourth generation. In the first is the "moderate drinker." In the second, excessive drinking and delirium. In the third, marked insanity with homicidal and suicidal impulses. In the fourth, hopeless insanity and dementia. The imbeciles and idiots come last, and the family is gone!

How shall we secure their birthright to the unborn? Read reliable books and journals that teach the truth about these things, and "the truth shall make you free" from your part of the sin against posterity. Give your moral support to the enactment and enforcement of legislation designed to prevent the procreation of the unfit, and the care of the feeble-minded. Avoid evil thoughts, such as anger, malice and envy, knowing that "thoughts are things" capable of exercising pre-natal influence on your child. Beg and pray, and, if necessary, demand that the father of your child be free from alcoholism and venereal poison. Teach your boys to live pure lives, that they may be fitted for fatherhood. Teach them that though "the lips of a strange woman drop as a honeycomb, her feet go down to death; her steps take hold on hell"; to physical death, to hell on earth, poisoning his system with disease, of which, though he may think he is cured, may still taint his blood so that he may send the wife whom he may marry to the operating table of the surgeon, or to an untimely grave, and may cause his children to be weaklings in body and mind, if not worse. Teach your daughters—what? To be pure? They are pure by nature and by precept and example of their mothers; but teach them to demand purity in those who are to be their husbands, the fathers of their children. Teach them to avoid the society of evil-minded women as they would a leper, and the whisky breath as the breath of pestilence.

Someone has said that to properly educate a child, we should begin with its grandmother. Mothers, you will be the grandmothers of your children's children; begin their education now by educating yourselves to properly discharge the most sacred of all duties—the duties of motherhood. Teach the duties of motherhood and fatherhood to your children and how they may discharge those duties, and your grandchildren will have a chance to secure their birthright—the right to be well born.

JOSEPHINE DANIEL.

---

THERE appeared recently in the *Dallas News* a splendid and startling article from the pen of Mrs. Calloway ("Pauline Periwinkle").

She cites us to some statistics compiled by Dr. Henry H. Goddard of Vineland, N. J., in which he proves conclusively that from one feeble-minded girl who was betrayed by a normal man sprang 480 descendants, only forty-six of whom were apparently normal. Thirty-six were illegitimates, thirty-three immoral, twenty-four confirmed confirmed alcoholics, and so on down the line of all the vices.

·Dr. Goddard makes the startling assertion that out of every one hundred school children three are feeble-minded, and that two of the three have inherited their feeble-mindedness and will transmit it; and what is yet more startling is this assertion: "These are not idiots and imbeciles, commonly recognized as such, but are among the good-looking, good-appearing boys and girls, who mingle freely with other children in our public schools." Mrs. Calloway further says: "According to statistics prepared by Dr. A. Caswell Ellis of our State University, there are estimated to be 12,988 feeble-minded persons in Texas. Of criminals studied in reformatories, 26 per cent are feeble-minded. At this rate, Texas has 900 criminals who have been made out of feeble-minded children—children not able to determine right from wrong." Texas has more than 1000 feeble-minded girls at large and unprotected. If not protected in special institutions, most feeble-minded girls become mothers. The penitentiary is, in Texas, the sole protection afforded society from feeble-minded fatherhood."

These facts and figures are appalling. What are we going to do about it? We are going to stop it. Who is going to stop it? You and I. How? There are three measures before the present Legislature which, if passed, will go far towards stopping the breeding of these poor unfortunates. One is a bill to provide a State institution for the feeble-minded; another is the sterilization of feeble-minded, lunatics, drunkards and criminals; the other seeks to prevent the marriage of any one without a health certificate.

Will you not sit down and write to *your* Senator and Representative and tell them, in plain language, that you expect them to vote for these measures and that you will take the pains to find out just how they do vote? It is wonderful how much can be accomplished by a plain, pointed letter. Try it, and see. You will be surprised—especially if you have a vote coming.

